# Prospects of Intraoperative Multimodal OCT Application in Patients with Acute Mesenteric Ischemia

**DOI:** 10.3390/diagnostics11040705

**Published:** 2021-04-15

**Authors:** Elena Kiseleva, Maxim Ryabkov, Mikhail Baleev, Evgeniya Bederina, Pavel Shilyagin, Alexander Moiseev, Vladimir Beschastnov, Ivan Romanov, Grigory Gelikonov, Natalia Gladkova

**Affiliations:** 1 Institute of Experimental Oncology and Biomedical Technologies, Privolzhsky Research Medical University, 10/1 Minin and Pozharsky Sq., 603950 Nizhny Novgorod, Russia; natalia.gladkova@gmail.com; 2 Thermal Injury Group, University Clinic, Privolzhsky Research Medical University, 18/1 Verkhnevolzhskaya Naberezhnaja, 603155 Nizhny Novgorod, Russia; maxim-ryabkov@yandex.ru; 3 City Clinical Hospital No.30, 85A Berezovskaya St., 605157 Nizhny Novgorod, Russia; baleev_ms@mail.ru (M.B.); vvb748@mail.ru (V.B.); post@hospital30.ru (I.R.); 4 The Department of Pathology, University Clinic, Privolzhsky Research Medical University, 18/1 Verkhnevolzhskaya Naberezhnaja, 603155 Nizhny Novgorod, Russia; genacrok@mail.ru; 5 Institute of Applied Physics of the RAS, 46 Ulyanova St., 603950 Nizhny Novgorod, Russia; paulo-s@mail.ru (P.S.); aleksandr.moiseev@gmail.com (A.M.); grgel@yahoo.com (G.G.)

**Keywords:** acute mesenteric ischemia (AMI), optical coherence tomography (OCT), optical coherence tomography angiography (OCTA), intraoperative diagnostics, gut vitality, image assessment

## Abstract

Introduction: Despite the introduction of increasingly multifaceted diagnostic techniques and the general advances in emergency abdominal and vascular surgery, the outcome of treatment of patients with acute impaired intestinal circulation remains unsatisfactory. The non-invasive and high-resolution technique of optical coherence tomography (OCT) can be used intraoperatively to assess intestine viability and associated conditions that frequently emerge under conditions of impaired blood circulation. This study aims to demonstrate the effectiveness of multimodal (MM) OCT for intraoperative diagnostics of both the microstructure (cross—polarization OCT mode) and microcirculation (OCT angiography mode) of the small intestine wall in patients with acute mesenteric ischemia (AMI). Methods and Participants: A total of 18 patients were enrolled in the study. Nine of them suffered from AMI in segments II-III of the superior mesenteric artery (AMI group), whereby the ischemic segments of the intestine were examined. Nine others were operated on for adenocarcinoma of the colon (control group), thus allowing areas of their normal small intestine to be examined for comparison. Data on the microstructure and microcirculation in the walls of the small intestine were obtained intraoperatively from the side of the serous membrane using the MM OCT system (IAP RAS, Russia) before bowel resection. The MM OCT data were compared with the results of histological examination. Results: The study finds that MM OCT visualized the damage to serosa, muscularis externa, and blood vessels localized in these layers in 100% of AMI cases. It also visualized the submucosa in 33.3% of AMI cases. The MM OCT images of non-ischemic (control group), viable ischemic, and necrotic small intestines (AMI group) differed significantly across stratification of the distinguishable layers, the severity of intermuscular fluid accumulations, and the type and density of the vasculature. Conclusion: The MM OCT diagnostic procedure optimally meets the requirements of emergency surgery. Data on the microstructure and microcirculation of the intestinal wall can be obtained simultaneously in real time without requiring contrast agent injections. The depth of visualization of the intestinal wall from the side of the serous membrane is sufficient to assess the volume of the affected tissues. However, the methodology for obtaining MM OCT data needs to be improved to minimize the motion artefacts generated in actual clinical conditions.

## 1. Introduction

Acute mesenteric ischemia (AMI) is a sudden interruption of the blood supply to a segment of the small intestine leading to ischemia, cellular damage, intestinal necrosis, and ultimately the patient’s demise if left untreated [1]. The disease accounts for approximately 0.2% of emergency hospital admissions [2]. Since 2020, the incidence has increased among adults [3,4,5] and children [6] due to the spread of COVID-19 infection.

AMI is life threatening for patients and a challenge for clinicians. Despite the introduction of increasingly advanced methods of diagnosis and treatment, the AMI mortality rate remains very high (45–85%) [7]. In most patients, ischemia would have lasted for more than 12 h prior to surgical intervention. Therefore, it is expected that large areas of necrosis would appear in the intestine. However, as the peripheral areas of the ischemic intestine continue to be partially supplied with blood from the collateral vessels, the damage is likely to be heterogeneous. One of the unresolved clinical quandaries is the objectivity of the intraoperative assessment of the viable sections of the intestine. Verification of regions of impaired blood circulation and tissue necrosis is essential for the selection of boundaries for bowel resection. Accurate verification reduces the risk of anastomotic leakage and helps prevent post-resection indigestion [8].

Currently, there are limited intraoperative tools available to surgeons in determining bowel viability. Surgeons mainly rely on a subjective assessment of the color and mobility of the intestine, and the presence of blood vessels pulsation. Thus, it is impossible to adequately assess the degree of ischemia-induced changes inside the intestinal wall. Studies on clinical estimation of bowel viability report an overall accuracy of 89% (positive predictive value 64%). In 46% of the cases, unnecessary bowel resection was reported [9]. Leaving non-viable intestine can lead to sepsis [10,11], whereas extensive resection of bowel can lead to short-bowel syndrome [12].

Various optical technologies could be used, allowing for direct visualization or quantification of the prevalence and severity of intestinal ischemia based on the intraoperative evaluation of microcirculation. These tools include Laser Doppler Flowmetry (LDF) and fluorescence imaging (FI). The former method is one of the pioneers in gut perfusion assessment [13,14,15,16,17], whereas the latter is widely used in contemporary clinical practice [18,19,20,21]. Note that the use of LDF has not become widespread in AMI affected patients, even though the method continues to be of interest in experimental studies of intestinal tissue damage following ischemia/reperfusion injury [22,23]. The last two years have witnessed an increase in the usage of LDF in combination with visible light spectroscopy in the gastroscopy of patients with chronic mesenteric ischemia [24,25]. A number of research groups expressed optimism regarding the great potential of FI using indocyanine green dye for routine use [19,20,26]. FI reduces operation time, and the technique has a laparoscopic potential. Even though FI is currently a method of choice in AMI, it is still not formally accepted as the standard of clinical care for intraoperative assessment of bowel viability [2,27]. Some researchers have found that FI is more effective relative to other available methods [19,20], whereas others have demonstrated its low significance [9,28,29].

Other optical methods that demonstrate potential in the detection of perfusion changes in the gut includes sidestream dark field (SDF) microscopy [30,31,32], incident dark field (IDF) imaging [33,34], and hyperspectral imaging (HSI) [35]. SDF and IDF are non-invasive microscopies for quantitative perfusion imaging, but have not been used in AMI. SDF demonstrates potential in the evaluation of perfusion of gastric tube [32] and colon [30,31] so that a reduction in anastomotic leakage could be accomplished. IDF with quantification related perfusion parameters was used in [33], only to determine angioarchitecture of the peritoneum, including capillaries. HSI is a novel technique for applications in intestinal ischemia. During a HSI imaging, false-color-images that represent different parameters of tissue oxygenation are generated [36]. Mehdorn at al. [35] demonstrates that HSI is able to discriminate tissue perfusion in AMI reliably and therefore might be helpful for resection. However, a more convincing assessment of the utility of HSI in AMI would require a cohort study with longitudinal and structured data.

Laser speckle contrast imaging (LSCI) is also a prospective technique for intraoperative assessment of intra-abdominal microcirculation in AMI [37,38]. However, so far it has only been used for the identification of ischemic areas on gastric tube reconstructions [39,40] and during colorectal surgery [41]. It is noteworthy that a special laparoscopic vision system integrating LSCI with a commercially available rigid laparoscope has already been developed and tested [42].

The abovementioned methods have their own advantages and limitations [43,44,45]. Furthermore, the intestine as an extraordinarily mobile research object also imposes additional constraints on the imaging tools and methods in terms of frequency, quality, and repeatability of the collected data. In particular, difficulties are caused by point research (LDF, SDF, IDF,) or non-contact (SDF, IDF, HIS, LSCI) methods.

Unfortunately, all of the above methods evaluate only intestinal perfusion and flowmetry, and ignore the assessment of structural tissue damage or the direct presence of necrosis.

Optical coherence tomography (OCT) angiography is a multimodal technology for the simultaneous visualization of the structure and microcirculation of the studied organ, and is most effectively used in ophthalmology [46]. Thus, it is a promising tool for the complex assessment of the intestinal wall damage caused by AMI. The experience of experimental and clinical application of OCT in gastroenterology indicates the possibility of the label-free imaging of the gut wall stratification with the resolution of 25 microns or less to the depth of 2 mm [47,48,49]. In the overwhelming majority of clinical studies using OCT, the gastrointestinal tract visualization has been carried out by endoscopic access, which has facilitated the assessment of mucous membranes of esophagus [50], stomach [51], and the initial part of small intestine and colon [52,53]. The main purpose of this procedure is to detect dysplastic epithelium, neoplasms, and to clarify the boundaries of tumor growth using only structural OCT images [54,55], or in combination with analysis of the mucosal microcirculation [54,56]. Methods are being developed for multivariate quantitative analysis of structural OCT images obtained from ex vivo gastric and intestinal samples of the mucous membranes to facilitate the use of such imaging of in vivo tissues for clinical applications in the future [57].

Due to the limited length of available probes for endoscopic intraluminal OCT, the small intestine distal to the duodenum and proximal to the cecum is still the least studied segment of the digestive tract using OCT [48]. A trans-serous transabdominal approach to an OCT study in a clinic would remove these restrictions and enable visualization of the serous and muscular membranes, and the network of subserous and submucous blood vessels in any segment of the small intestine. This is very important for diagnosing the prevalence of AMI. In [58], OCT was used to determine the degree of perfusion of the anastomosis of the esophagus by the stomach wall during reconstructive surgery. However, it used OCT from the outer layer of the gastrointestinal tract. An OCT picture of ischemic damage to the wall of the small intestine in humans with AMI has not been previously obtained or assessed. We set out to test whether multimodal (MM) OCT, which has proven its effectiveness in studies of AMI models in laboratory animals [49,59], is effective here. This would enable us to obtain objective data on the state of the human intestinal wall with AMI in a clinical situation.

The purpose of this study is to demonstrate the effectiveness and prospects of trans-serous MM OCT in intraoperative diagnosis of the microstructure and microcirculation of the small intestine wall in AMI patients.

## 2. Materials and Methods

### 2.1. Preparatory Animal Study

Porcine intestine was selected for the testing and probe positioning during MM OCT imaging. In terms of structure and thickness of the wall, this is nearest to the intestines of our human experimental subjects. It is known that many physiological movements of the gut, and of the organism as a whole, can lead to significant artefacts in the OCT data. Therefore, the stabilization of the probe and the object under study is critical for obtaining high-quality structural OCT, and especially OCT angiography (OCTA) [46,60] images.

Normal small intestine of a laboratory miniature pig was imaged with MM OCT before the clinical study. One Wiesenau male miniature pig, 4 months old, weighing 37 kg, was used. The animal was kept in accordance with the rules adopted by the European Convention for the Protection of Vertebrate Animals used for Experimental and Other Scientific Purposes (Strasbourg, 1986). The small intestine was accessed through a layered midline laparotomy under combined (gas and intravenous) anesthesia, with continuous monitoring of the electrocardiogram, blood pressure, and body temperature.

### 2.2. Patients

The controlled single-center observational study included 18 patients (10 men and 8 women). All of them were operated in 2019–2020 for their abdominal surgical pathology. Target group included 9 patients who were hospitalized and urgently operated for AMI. Inclusion criteria: AMI as a result of thrombosis; embolism of arteries associated with segments II–III of the superior mesenteric artery with acute ischemia of the ileum. Exclusion criteria: Thrombosis or embolism in segment I of the superior mesenteric artery; the use of vasopressors in the perioperative period to relieve hypotension. The diagnosis of AMI in 5 out of the 9 patients was established before laparotomy on the basis of the clinical picture and computed tomography data. The same diagnosis was made in 2 out of the 9 patients on the basis of clinical picture and diagnostic laparoscopy data. Finally, diagnosis of AMI in the residual 2 patients were made only after emergency laparotomy due to the clinical picture of acute surgical abdomen of unknown etiology. Preoperative examination of 4 patients (2 women, 2 men) included into the target group revealed chronic cardiac arrhythmias in the forms of atrial fibrillation (*n* = 2), ventricular tachyarrhythmia (*n* = 1), and bundle branch block (*n* = 1). Fragments of emboli localized in the II and III segments of the superior mesenteric artery were found in histologic specimens obtained from 3 of these patients during resection of the necrotic intestine with the mesentery. Another 4 patients from the target group had atherosclerotic changes in mesenteric vessels even though only 1 out of 9 patients reported about the signs of chronic mesenteric ischemia in the form of postprandial pain in the past 4 years. The volume of surgery in the target group involved resection of a non-viable part of the small intestine, the boundaries of which were determined by visual-palpation criteria [2].

Control group included 9 patients who were on planned treatment for adenocarcinoma of the colon (T3—4N0M0), without intestinal obstruction, bleeding, perforation, or other intra-abdominal complications. The scope of the operation in this group was right-sided hemicolectomy with resection of the terminal section of the ileum. Since the blood supply and structure of the ileal section in these patients were not impaired in any way (the distance from the tumor was more than 60 cm), the parameters of the wall of this section of the intestine were diagnosed as normal. Chronic cardiac arrhythmia was detected in 2 out of 9 patients in the control group.

Some aspects regarding patient characteristics are presented in Table 1. With a tendency towards a higher median body mass index in patients in the control group in comparison to the target group (27.3 vs. 24.8, the differences are not statistically significant, *p* = 0.113, Mann–Whitney test), the decrease in body weight by more than 10% within 6 months before the operation in the control group was noted in 4 patients, and in the target group—only in 1 patient. The median age, body mass index (according to the Mann–Whitney test), and the incidence of concomitant diseases (Fisher’s exact test) in the patients of the studied groups had no statistically significant differences (in all cases, *p* > 0.5) (Table 1).

Preoperative and postoperative therapies and surgical interventions were performed in accordance with the established protocols and standards of medical care [2]. The study was carried out in accordance with international ethical principles and the regulatory requirements of Russian Federation. All patients received an exhaustive explanation of the purpose, algorithm, and possible consequences of intraoperative therapeutic and diagnostic manipulations, after which they gave informed consent for the procedures to be performed. This study was approved by the review board of the Privolzhsky Research Medical University (Protocol #17 from 10 November 2019). The research was carried out within the framework of the RSF project #19-75-10096, agreement 19-75-10096 from 9 August 2019.

### 2.3. MM OCT Device

**Figure 1:**
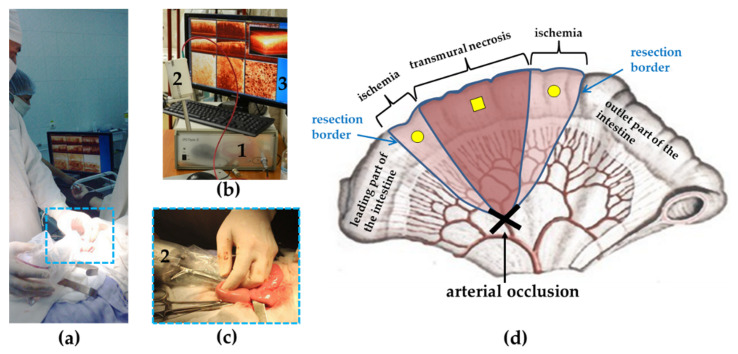
The procedure for intraoperative small intestine visualization using multimodal optical coherence tomography (MM OCT). (**a**) General overview of the trans-serous MM OCT study; (**b**) MM OCT system (1) with probe (2) and an example of data set acquired after tissue scanning (3); (**c**) the probe (2) in a sterile sheath is placed on the surface of the normal ileum; (**d**) diagram of the MM OCT study of ischemic bowel: square mark—point of the OCT probe localization on the anesthetized part of the intestine, round marks—points from which MM OCT images were obtained from the ischemic areas of the intestine to be resected.

A custom-made spectral-domain MM OCT setup (Figure 1b) was used in the study (Institute of Applied Physics of the RAS, Russia) [61] for contact intraoperative visualization of the intestinal walls of the patients (Figure 1a,c). The setup combines traditional structural OCT as well as cross-polarization and angiography modes [62].

The radiation source in the device is a superluminescent diode with a central wavelength of 1310 nm. Note that the longitudinal resolution is 10 µm, the lateral resolution is 15 µm, while the scanning depth in air is ~1.7 mm, and the scanning rate is 20,000 A-scans per second. Continuous 2D scanning is performed along the fast axis (512 spectral measurements, 256 spectral A-scans) and along the slow axis (1024 B-scans), which are aggregated in a 3D image. As a result, 2D depth-wise images and en-face images are available for display and evaluation. The use of circularly polarized light allows for the construction of two types of structural images. The first image type reflects the degree of backscattering of light from all those tissue components capable of backscattering probe radiation (image in co-polarization). In contrast, the second image type shows the result of only the cross-scattering from fibrous components (mainly collagen, and to a lesser extent muscle fibers) under orthogonal orientation of the polarization vector of the probe radiation (image in cross-polarization). Both images are combined into one cross-polarization (CP) OCT image.

The angiography processing is described in detail in [62]. As a brief summary, the angiography data is extracted from the 3D OCT volume, acquired by continuous scanning, i.e., each lateral position in the volume was scanned only once. To preserve high correlation between neighboring scans for the tissue bulk, the scanning step was chosen to be about ¼ of the scanning beam width. The perfused blood vessels were contrasted from the stationary surrounding tissue by high-frequency filtration in the signal domain along the slow axis. In practice, this implies that the application of predefined coefficients to every 7 B-scans neighboring along the slow one yields a single angiography cross-section frame. This allows real-time visualization of the vascular cross-sections during the OCT data acquisition, providing necessary feedback for the user.

The vascular imaging includes an en-face OCTA image constructed during the process of data acquisition. Places where blood is in a stationary state are not visualized. For OCT angiographic analysis, each obtained 3D microvascular network was converted to 2D image with Maximum Intensity Projection (MIP) by projecting the desired imaged depth onto the plane. The OCTA images shown in the study were not post-processed to remove motion artefacts, as the purpose of using OCTA for microvascular network visualization in the intestines of patients in this study was to demonstrate the applicability of the method to real clinical conditions, rather than to present a scrupulous quantitative assessment of the vascular network.

It takes 26 s to acquire one set of images. This includes a 3D image, 2D depth-wise images in different planes, 2D en-face images, and an OCTA image of the intestine (Figure 1b, number 3).

As a design feature, the MM OCT device included an elongated cylindrical contact probe 15 cm long and 1 cm in diameter (Figure 1b, number 2). In an open operation with a laparotomic approach, such a probe design provided visualization in every event of the small intestine segments requiring examination. During scanning, the probe was either handheld by the surgeon, or was rigidly fixed onto a custom-made holder (KARL STORZ SE & Co. KG, Tuttlingen, Germany).

### 2.4. MM OCT Examination of the Small Intestine

MM OCT data were obtained by trans-serous access during laparotomy (in porcine) and surgery (in patients). The optical probe was placed in contact with the lateral surface of the intestine, at a point equidistant from the mesenteric and antimesenteric edges (Figure 1d).

For the porcine tests, twelve 3D MM OCT images were obtained from the small intestine of the pig. In particular, 3 volumetric images were recorded from each of the four zones of the intestine for the purpose of histological examination.

In the studied groups of patients, a total of 60 3D images were obtained. Among them, 45 images contained the least number of artefacts and thus were selected for further analysis. In AMI group, a total number of 3D images was 27, and they were obtained from three locations. One of these locations was in the center of the affected segment, where the intestine had a cyanotic color, there was no shine to the peritoneum, the mesenteric vessels did not pulsate and there was no observable peristalsis. Nine volumetric images were reordered from this point. At the other two locations (18 volumetric images)—at the periphery of the ischemic area (left and right), the color and luster of the intestine were changed and peristalsis was weakened, but no visual signs of transmural necrosis were detected (Figure 1d). These parts of the intestine also had to be removed. Thus, all 27 areas of the MM OCT study were within the resection and were subject to subsequent histological confirmation. In the control group prior to hemicolectomy, 18 3D datasets were obtained from 2 locations of the normal ileum: At the proposed border of resection for the possibility of comparison with histological data, and at a point located 25 ± 2 cm proximal to the ileocecal angle from the zone corresponding to a similar area in the case of the ischemic small intestine. No histological verification of this section of the small intestine was required due to the similarity in the histological structure of the two studied areas of the control group.

For the assessment of structural CP OCT images from each sampled volume, several (2–4) representative 2D cross-sectional and 2D en-face images at the depth of different intestinal wall layers were saved and assessed. In terminology, cross-sectional CP OCT images will be called B-scans, as is customary in OCT technology [63]. During visual analysis of the CP OCT images, the following two characteristics of the intestinal wall microstructure were assessed. First, the number of intestinal wall layers. Second, the presence and severity of signs of intermuscular edema. The former could be differentiated on the B-scans whereas the latter was assessed both in the B-scans and in the en-face images.

Microcirculation was analyzed in 2D en-face OCTA images (*n* = 45). Visual assessment of the OCTA images included criteria for the presence/absence of large blood vessels, of branched networks or of individual vessels, and the presence/absence of a microcapillary network.

### 2.5. Histological Study

After MM OCT imaging of the intestinal wall the scanned area was marked with histological ink. Following resection in line with the surgery plan, samples were taken from the removed parts of the intestine for histological evaluation. The specimen was fixed in 10% formalin for 48 h and the histological sections were prepared through the marked areas, so that their planes corresponded to the depth-wise CP OCT images. Hematoxylin and eosin (H&E) staining was used. A histopathologist blinded to the intestinal condition evaluated the histological samples for the presence of edema in the serous and muscle layers and inflammation. The said histopathologist also assessed the state of the blood vessels and classified the samples as either “normal” “with a predominance of ischemic tissue damage” (necrosis of individual villi was allowed) or “widespread tissue necrosis”. Digital photographs were obtained in transmitted light with a Leica DM2500 DFC (Leica Microsystems, Germany) microscope, equipped with a digital camera. The results of the histopathology were compared with the corresponding CP OCT and OCTA-based findings.

### 2.6. Statistical Analysis

For statistical data processing, the program IBM SPSS Statistics software, V20. (IBM Corporation, Somers, NY, USA) was used. The results are expressed as the Me [Q1; Q3], where Me is the median of the analyzed parameter and [Q1; Q3] are the 25th and 75th percentile values, respectively. The Mann–Whitney U-test and Fisher’s exact test were used to compare quantitative and qualitative data, respectively. *n*—the volume of the analyzed subgroup, *p*—the magnitude of the statistical significance of the differences. The critical value of the significance level was set to 5% (*p* ≤ 0.05).

## 3. Results

### 3.1. MM OCT Study of the Normal Pig Intestine

Porcine small intestine was visualized in vivo with the MM OCT system to establish the methodology. Furthermore, it presented a representation of the intestinal wall microstructure and microcirculation features from the direction of the serous membrane, which is essential for comparison with the subsequent human studies.

**Figure 2:**
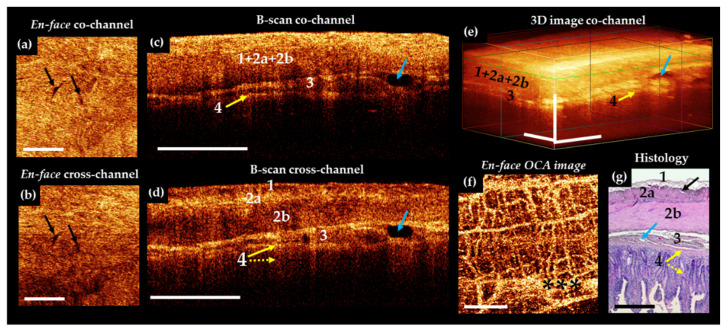
Representative trans-serosal MM OCT images (**a**–**f**) and corresponding histology (**g**) of normal small intestine of a pig. In the B-scans cross—channel (**d**) all 4 layers—serous membrane (1), muscularis externa (longitudinal muscular layer 2a and circular muscular layer 2b), submucosa (3) with blood and lymphatic vessels (blue arrows) and mucosa (4) (in particular, muscularis mucosa (yellow solid arrow) and adjacent to it the lamina propria (yellow dotted arrow)) are more clearly resolved, while in the co-channel not all anatomical layers are optically separated (**c**). (**a,b**) En-face OCT images at the level of the longitudinal muscular layer (green horizontal line in (**e**)). Intermuscular spaces look like slits and there are few of them (black arrows). In the OCTA image (**f**), the network of blood vessels is dense and branched. Large and small blood vessels are visualized; the background of the image is light due to a dense network of a functioning capillary network. (**g**) Histological specimens stained with H&E. Asterisks in (**f**) indicate artefact of motion. Scale bars in CP OCT and OCTA images 1 mm, in histology 0.5 mm.

Several types of artefacts may be present in OCT [64] and OCTA images [65]. In the overwhelming majority of cases, images contain 1 or 2 wide horizontal stripes (Figure 2f, black asterisks)—a consequence of the subject’s breathing movements or series of thin, often spaced horizontal lines—the result of heartbeat (pulse waves) [62]. They are most noticeable in the OCTA images. The bowel movement itself is best seen in the structural en-face images as a shift of the structures to the left and right with the formation of wavy patterns in the picture.

In the process of recording the MM OCT data, it was found that to minimize motion artefacts in the images, immobility of the tissue relative to the probe surface is required. At the same time, to preserve the true picture of microcirculation, it is important to prevent tissue compression. The probe approaching the intestine should be under the OCT control until a full contact with the entire surface is achieved. After that, the probe must be rigidly fixed, for example with a custom-made holder (we used the holder from KARL STORZ SE & Co. KG, Tuttlingen, Germany). If the study is carried out without a holder, it is optimal to position the probe and the intestine in a horizontal plane, with a temporary removal of the examined intestinal loop from the laparotomic wound. However, despite all efforts, it was almost impossible to completely eliminate all movements and get a real-time image completely free of any artefacts.

The analysis of the porcine structural OCT images in co- and cross-polarization channels (Figure 2c–e) based on their comparison with the results of the histological evaluation (Figure 2f) revealed that the depth of visualization of the intestinal wall depends on the muscularis propria and submucosal thickness, but in general, all the layers are potentially clearly visible, including the lamina propria of the mucosa adjacent to the muscularis mucosa. The structure of the villi themselves are indistinguishable on the structural CP OCT images due to the strong attenuation of the OCT signal at a depth of about 1.3 mm.

In the B-scans co-polarization channels, as a rule, the serous and muscularis propria are characterized by a high degree of scattering, the intensity of which decreases with depth (Figure 2c, numbers 1,2a,2b). Vertical heterogeneity is created by shadows from the blood vessels of the serous and muscle layers. The vessels themselves are almost indistinguishable on the structural images due to insufficient contrast with the surrounding tissues. The submucosal layer is perfectly visible due to the presence of connective tissue (Figure 2g, number 3) and muscularis mucosa (Figure 2g, number 4, yellow arrow), being visualized as two horizontal stripes (Figure 2c,d, numbers 3 and 4, yellow arrow). Large and small blood and lymphatic (Figure 2c,d, blue arrows) vessels are located in submucosa. It has been established that the cross-polarization image (Figure 2e) provides more information about the layers of the intestinal wall, allowing separate detection of the serosa (Figure 2e, number 1) and the two muscular layers. Note that the mutually perpendicular arrangement of the muscle layers and the dense parallel packing of the muscle bundles within them leads to polarization effects, as a result of which the outer muscle layer looks brighter than the inner one (Figure 2e, numbers 2 and 3). Similarly, the submucosa (Figure 2e, number 4) is clearly evident due to the sensitivity of polarization to the properties of the collagen fibers forming the connective tissue of this layer. In the en-face OCT images at the level of the outer muscle layer, single slit-like spaces are noticeable (Figure 2a,d, white arrows), which correspond to intermuscular spaces (Figure 2g, black arrow).

In the OCTA images of the porcine intestine, a dense, branched network of blood vessels can be observed (Figure 2f). The contours of the large and medium-sized blood vessels are clearly traced, and numerous functioning capillaries create a bright background throughout the image. By varying the depth of visualization of the vasculature and comparing it with the level at which it is visible in the conjugated en-face images, it is possible to analyze the microcirculation in the different layers of the intestinal wall.

After the successful conclusion of the porcine intestine study, we are now prepared for MM OCT data acquisition from the human intestine in the operating room. An algorithm of actions (methodology) as described above was developed for the upcoming study of the human intestine.

### 3.2. CP OCT Study of Normal and Ischemic Human Intestine

In vivo MM OCT study of small intestine, performed by trans-serous access, allowed non-invasive visualization of the intestinal wall in all patients to a depth of 1500 to 1800 µm and verification of its layer-by-layer structure. The optical equivalents of the layers of the intestinal wall and the intramural blood vessels were determined by comparing the CP OCT and OCTA images with the histological sections precisely made in the same area.

**Figure 3:**
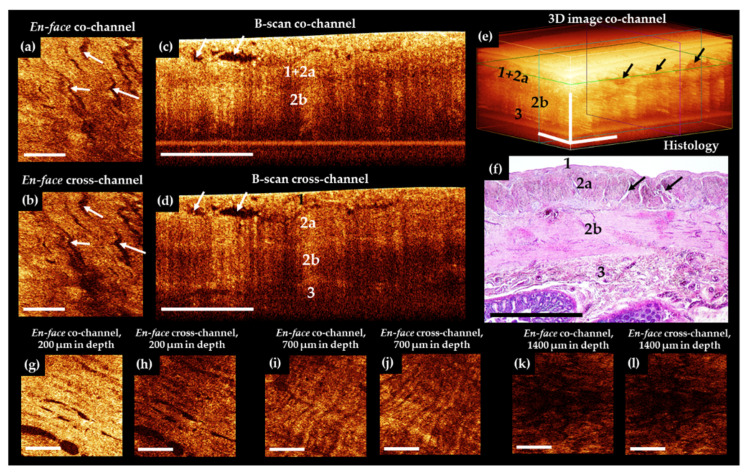
Representative trans-serosal CP OCT images of normal human ileum. In the B-scan cross-channel (**d**) the serous membrane (1), muscularis externa (longitudinal muscular layer 2a and circular muscular layer 2b) and the boundary with submucosa (3) are more clearly resolved, while in the co-channel, not all anatomical layers are optically separated (**c**,**e**). (**a**,**b**) En-face OCT images at the level of the longitudinal muscular layer (green horizontal line in (**e**)). Intermuscular spaces ((**f**), black arrows) look like slits in CP OCT images ((**a**–**d**), white arrows; (**e**), black arrows) and they are located within the outer muscular layer. (**f**) Corresponding histological image, H&E staining. In some cases, a clear change in the direction of muscle bundles in different layers (**g**–**j**) and a characteristic pattern of the lamina propria (**k**,**l**) immediately under the muscularis mucosa can be observed. Scale bars 1 mm.

Representative CP OCT images of normal human ileum (patients of control group) are shown in Figure 3.

The results of comparison of OCT B-scans with histological samples revealed that in most cases, the intestinal wall was visualized in depth up to the submucosa. The serous membrane 40–100 µm thick—the first highly scattered layer—practically merges with the underlying muscle layer, but is marginally better distinguishable in the cross-channel (Figure 3d, number 1). A distinctive feature of the outer muscle layer (Figure 3c–e, number 2a) is its heterogeneity due to the presence of numerous slit-like spaces, which are clearly visible in the en-face images (Figure 3a,b, white arrows). They indicate the location and orientation of the muscle bundles (Figure 3f, black arrows). This layer is up to 400 µm thick. In the co- and cross-channel B-scans, the intermuscular spaces are contrastingly visualized as negative. Note that the separately located inclusions with a predominantly horizontal orientation (Figure 3c,d, arrows) are due to tissue compression by the probe, whereas, on the corresponding histological specimen, these slit spaces are located at different angles to the surface (Figure 3f, black arrows). The inner (circular) layer of muscle (Figure 3c–e, number 2b) is localized at depth 450–1100 µm, with the zone of transition of the outer muscle layer to the inner one being better defined in the B-scans cross-channel (Figure 3d). This is due to a local increase in the OCT signal intensity and the change of direction of the muscle fibers. The change of direction of the muscle fibers between the different muscle layers (see Figure 3g–j) can be used during the search for blood vessels or layer transition. In some cases, submucosa and very rarely part of the mucous membrane (muscularis mucosa and the adjacent to it lamina propria, Figure 3k,l) were visualized. It is possible to visualize in cases of naturally reduced thickness of the muscle and submucosal layers. For example, when the intestinal wall is stretched by a gas bubble located in the lumen of an organ.

Of 18 CP OCT images of normal human small intestine, the serous membrane and muscularis externa were visualized in 18 (100%) out of 18 cases. The submucosa were visualized in 12 (66.7%) out of 18 of cases. Finally, the mucosa were visualized in 2 (11.1%) out of 18.

**Figure 4:**
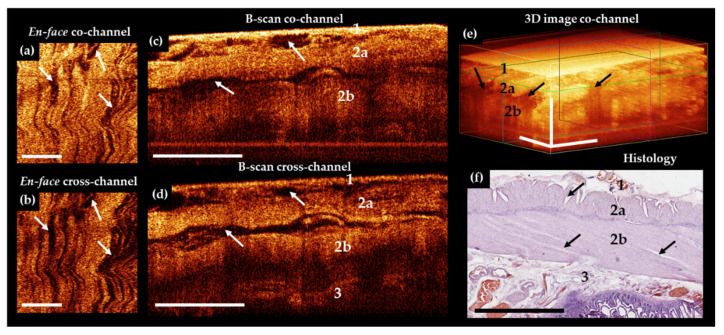
Representative trans-serosal CP OCT images of ischemic ileum without necrosis. (**a**,**d**) In the B-scans co–**c**) and cross–(**d**) channels and in 3D OCT image (**e**) the general architecture of the serous membrane (1) and muscularis externa (outer longitudinal—2a and inner circular—2b layer of smooth muscle) are clearly resolved with the extensive accumulation of intermuscular fluid (white and black arrows) within each layer. The boundary between the circular muscular layer (2b) and the submucosa (3) is more clearly visible in the B-scan cross—image (**d**). (**a**,**b**) En-face OCT images at the level of the longitudinal muscular layer (green horizontal line in (**e**)), intermuscular spaces (white arrows) are longer and wider than in norm (Figure 3a,b). (**f**) Corresponding histological image, H&E staining. Black arrows indicate intermuscular edema. Scale bars 1 mm.

Typical CP OCT images of ischemic, but viable, small intestine of the patients in AMI group are demonstrated in Figure 4.

Bowel wall thickening was a common finding in the OCT images. Despite external signs of tissue nonviability, in the CP OCT images the general architecture of the outer two layers (serosa and muscularis externa) was preserved, but a large number of well-contoured negative cavities (intermuscular edema) were present in images within all the imaged layers (Figure 4a–d, arrows). Again, in the cross-channel (Figure 4e), the boundary between the circular muscle layer (number 2b) and the submucosa (number 3) is more clearly visible. According to histological analysis, in the adjacent segments of the intestine where the blood supply was partially preserved, no transmural necrosis was found (Figure 4f). The manifestations of ischemia in samples of damaged but viable intestine were detected with different frequencies. In particular, pronounced edema of the serous and muscle layers in 14 out of 18 cases, and moderate edema in the residual 4 cases. Thickening shortening of villi and desquamation of enterocytes were also observed in 7 out of 18 cases. Necrosis of the submucosa and the muscle layer was not observed. Infiltration of the tissue of the mucous membrane with neutrophils and lymphocytes was also a manifestation of ischemia. It was noted in 14 intestinal samples out of 18. The spread of cellular infiltration into the submucosa and into the muscle layer was noted in 7 cases.

Of 18 CP OCT images of the ischemic small intestine, serous membrane and muscularis externa were visualized in all 18 (100%) cases, while submucosa was visible only in 6 (33.3%) cases. The latter is due to the overall bowel wall thickening.

**Figure 5:**
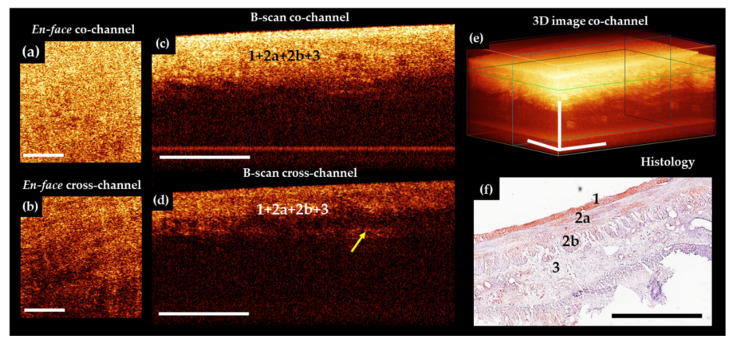
Representative trans-serosal CP OCT images of ileum with transmural ischemic necrosis. In the B-scans co– (**c**) and cross– (**d**) channels and in 3D OCT image (**e**) the depth of tissue visualization is decreased significantly and individual layers of the serous membrane (1), outer longitudinal muscular layer (2a), and inner circular muscular layer (2b) are not distinguishable due to the developed necrosis in all layers of the intestinal wall (**f**). In the B-scans cross-channel (**d**) in the lower part of the inner muscle layer, the signal is noticeably reduced, and the transition from this layer to underlying tissue of the submucosa (3) can be detected. (**a**,**b**) En-face OCT images at the level of the longitudinal muscle layer (green horizontal line in (**e**)), demarcated from the surrounding tissue fluid accumulation is not observed. (**f**) Corresponding histological image, H&E staining. Scale bars 1 mm.

In a subgroup of AMI patients with histologically confirmed necrosis of most of the intestinal wall, the CP OCT images (Figure 5a–e) appeared structureless, and in most cases it was not possible to establish any different layers within the intestinal wall. 

However, in 4 out of 9 cases, the serosa was distinguishable in B-scans co-channel owing to abnormally high scattering of the serous membrane after its impregnation with blood. In 2 (22.2%) out of 9 cases, boundaries between the inner muscle layer and the submucosa were detectable due to a sharp decrease in the signal in the inner muscle layer under cross-channel (Figure 5d, number 3).

Histological analysis of the intestine with macroscopic signs of transmural necrosis revealed that the thickness of the serous membrane and the two muscle layers was greatly reduced (Figure 5f). All the layers were damaged. Subtotal destruction of villi with desquamation of the enterocytes (in 9 out of 9 cases) was identified in mucosa. Infiltration of preserved fragments of the mucosa and the submucosal layer with neutrophils and eosinophils were observed in 9 out of 9 cases. Furthermore, we observed vascular thrombosis and subtotal vasculature in 8 out of 9 cases, hemorrhagic impregnation of areas of the serous and muscularis propria in 6 out of 9 cases, and foci of lysis of smooth muscle tissue in 4 out of 9 cases.

Thus, the structural OCT conducted via the co- and cross-polarization channels found that:

(1) The serous membrane and the outer and internal muscle layers could be visualized in the normal intestine. Furthermore, they could be differentiated from the underlying layers in all observations. In particular, visualization of the submucosa and its differentiation from the muscle layer was feasible in 12 out of 18 cases. The former could be differentiated from the mucosa in 2 out of 18 cases.

(2) The ischemic bowel differed from the normal one because of the accumulation of intermuscular fluid not only under the serous layer, but also between the bundles of both muscular layers. In contrast to the visualization of necrotic tissues, where the wall stratification was not observed with MM OCT, in these areas the structure of the outer layer was visible confirming their anatomical integrity. The serous membrane and muscle layers are visualized and can be clearly differentiated in all OCT images. However, it was possible to differentiate the submucosa only in 6 out of 18 cases.

(3) The necrotic wall of the small intestine appeared structureless in co-channel. In cross-channel, the stratification of layers appeared disturbed. Nevertheless, serosa was distinguishable in 4 out of 9 cases and inner muscular layer from the submucosa in 2 out of 18 cases. No delimited fluid accumulations were observed in the necrotic intestine.

### 3.3. OCT Study of Intramural Microcirculation in Normal and Ischemic Human Intestine

In order to exclude the influence of hemodynamic and blood composition parameters among the patients on the quality of visualization of blood vessels using the OCTA method, we compared these main indicators in the groups under study. The results are presented in Table 2. There were no statistically significant differences in the analyzed parameters between AMI and control groups (in all cases *p* > 0.5).

The influence of the other (except of hemodynamic parameters) most significant factors capable of changing the picture of the microvasculature in the abdominal cavity, but not directly related to the mechanisms of intestinal ischemia development, was taken into account and was minimized. In particular, during the operation, the level of intra-abdominal pressure and coronary perfusion pressure were routinely monitored in all patients. Both parameters remained within the normal range. The administration of vasopressors during the operation in time of the MM OCT study was not carried out. There were no signs of peritonitis (acute intestinal obstruction according to the examination of the abdominal cavity, culture of microflora from the abdominal cavity) in the target and control groups. The external factors (such as muscle tremor of the surgeon’s hand and vibrations from the operating room equipment) were minimized as much as possible and were also taken into account during the analysis of the OCTA images. The appearance of motion artifacts is very different from the shape of blood vessels. This allows if not the suppression of micro-movement influences (which is possible due to additional digital processing in some cases), but at least appropriate identification of them in the OCTA images.

**Figure 6:**
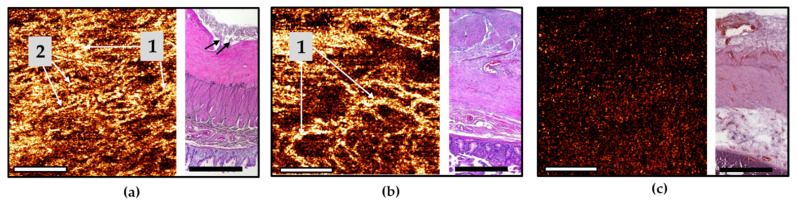
En-face OCTA images of the human small intestine in norm and ischemia with the corresponding histology. (**a**) A network of blood vessels of different diameters in the normal intestine wall; (**b**) ischemic bowel without extensive necrosis—noticeable rarefaction of the vasculature and in some areas the background formed by microvessels disappeared; (**c**) ischemic bowel with transmural necrosis. OCTA images in (**a**,**b**) demonstrate the microvascular network from the tissue surface down to a depth of 500 µm, which corresponds to the blood vessels of the serosal and longitudinal muscular layers, in (**c**) the blood vessels are not visible, only the signal noise (snow-like spots) is present. Black arrows in histological image (**a**) indicate non-damaged sub serous blood vessels. 1—intramural blood vessels, 2—light background from the microvessels. Histological specimens are stained with H&E. Scale bars 1 mm.

OCTA examination of the patient’s normal intestine and the parts with ischemia but without necrosis showed that, as a rule, the main part of the perfused vessels could be visualized up to a depth of 300 μm, which corresponds to the blood vessels of the serosal and longitudinal muscular layers. In the OCTA images, superficially located blood vessels had the appearance of linear branched structures, while the perfused microvascular network created a bright background. Branched vessels of different diameters were visualized in the wall of the non-ischemic intestine (control group) (Figure 6a).

The vasculature was visible in all the patients of the control group, despite the fact that motion artefacts (bright thin and wide horizontal stripes) were inevitably present on the OCTA images (Figure 6a). Histological examination confirmed the presence of non-damaged blood vessels in the serous membrane, with large vessels penetrating the peritoneum and spreading into the subserous space, while there were also numerous arterioles and venules penetrating the muscle layers of the intestinal wall. No ischemic injuries were found in this group of patients (Figure 6a).

In patients with AMI, the optical equivalents of the vessels differed significantly in areas with irreversible changes (necrosis) and in viable ischemic tissue. In areas with irreversible ischemic injury and necrosis, blood vessels either could not be visualized at all on the OCTA images or only showed as single vessels, while the background of the angiogram was homogeneous and predominantly dark (Figure 6c). In areas with histologically confirmed viable ischemic tissue, the OCTA images revealed a few blood vessels of different diameters, but they did not form a continuous network, and some of them looked interrupted. The background of the OCTA image was predominantly dark due to the lack of blood perfusion in vessel lumen (capillaries, small arterioles, and venules), which is a prerequisite condition for visualization by the OCTA method (Figure 6b). The histological picture in this case included ischemic damage to the vascular system in the form of the presence of a plethora of mostly large blood vessels in stasis with sludge in some of the vessels, while almost all the capillaries were in stasis (Figure 6b). Therefore, the capillaries were not visualized in the OCTA images.

In accordance with the approaches [66,67] and the experience of our group with experimental studies [15], a full (i.e., effective) intramural microcirculation requires the presence of blood flow in two components of the microvascular network: 1—in the intramural arteries that anastomose with the vessels of adjacent sections of the intestine; 2—in the microvascular network of the submucosa. We found that both of these conditions are met in the normal state. In ischemia without necrosis, the intramural arteries could be visualized, but did not form a continuous network; at the level of the submucosal layer, the vessels were distinguishable in (33.3%) 6 out of 18 cases. This fact suggests that in this case, ischemia is of a non-occlusive nature. Measures aimed at enhancing the bloodstream could transition a situation with ischemia towards an increase in the number of perfused vessels, starting the processes of restoring the structural integrity of the damaged sections of the intestine. In cases of ischemia with transmural necrosis, no perfused large or small blood vessels could be observed, indicating a completely impaired blood flow in these areas. Visually, they could also be recognized as non-viable and were therefore subject to mandatory resection.

A comprehensive analysis of MM OCT of the small intestine has thus revealed significant differences in the qualitative characteristics of its microstructure and microcirculation in patients with normal intestines and those with AMI. Table 3 summarizes these data.

## 4. Discussion

The technologies used for accurate topical diagnosis of ischemia in the organs of the digestive tract are focused on identifying qualitative and quantitative parameters of blood flow during surgery and predicting the risk of ischemic necrosis. For emergency surgery in cases of AMI, the possibility of simultaneous visualization of the perfused microvessels along with assessing the state of the layers of the intestinal wall at each point of interest is particularly valuable for the surgeon. Multidetector computed tomography with intravenous contrast is the main method of screening diagnostics and preoperative examination of a patient with suspected AMI [8], but it is not suitable for intraoperative use. The method is able to verify the occlusion of arteries (as a rule, extramural) with a diameter of more than 1 mm [68], but are not suitable for accurate mapping or diagnosis of blood flow in intramural vessels. During surgery, LDF, FI, SDF, IDF, LSCI, HIS, and OCTA can be helpful to determine the intestine viability and clarify the exact border of bowel resection [43,44,45,69]. LDF, FI, SDF, IDF, LSCI, HIS techniques obtain information about perfusion/blood flow and only OCTA assesses bowel microvessels and microstructure simultaneously [49].

The suitability of each method in the spectrum of diagnostic technologies for intestinal ischemia is determined by its resolution, visualization depth, dimensions of the field of view, and the ability to quantify data [43,44,45,69]:

(1) The resolution should be sufficient to detect microvessels and tissue structural features. Image resolution of IDF (3.3 µm), LSCI (10 µm) and OCTA (5–15 µm) is greater than in SDF (30–50 µm) and FI (450 µm), which is enough for capillary visualization. Conventional LDF offers no image, only measurements in digital format.

(2) The imaging depth should be sufficient to reveal structural or blood flow changes not only in the superficial layers (serous membrane and muscularis externa), but also in a deeply localized tissue (the submucosa and mucous layers), from where necrosis begins. Maximal imaging depth of IDF, SDF, and LDF is about the same and is 0.5 mm, which is rather small for a complete picture of gut microcirculation. Other methods allow seeing deep into the tissue up to 1 mm with LSCI, 1–1.5 mm with FI, 1.5–2 mm with OCTA, and 1–6 mm with HIS, therefore they are better suited for diagnostic purposes.

(3) Considering that the gut has a large surface area, the size of the field of view of the optical method is also important when it is inspected for the boundaries of the viable tissues. From this position, the FI method has an undeniable advantage. It allows assessing the entire length of the intestine at once. Field of view of LSCI and HIS is measured from a few square centimeters to several tens of square centimeters. It is also sufficient for assessing large bowel surfaces. OCTA visualizes from 9–10 mm^2^ to 100 mm^2^, while IDF and SFD have the smallest field of view (1–2 mm^2^).

(4) Quantification of data objectifies them, allowing for finding the exact criteria for separating two states. The IDF and SFD methods have an advantage in quantification of vascular networks: At least 4 microcirculatory parameters can be calculated (total and perfused vessel density, the proportion of perfused vessels, the microvascular flow index). Other methods (OCTA, LDF, LSCI, and HIS) also have a quantitative expression of blood perfusion data using already developed and tested methods, while the first attempts at quantifying the fluorescence signal were made for FI [29].

In conclusion, a comparison of the main characteristics of optical methods showed that each has certain advantages and limitations. The strengths of a specific technology are used to address a specific clinical problem. In the future some of these technologies could have complementary usage. For instance, OCTA lack of imaging range could be addressed by complementary utilization of LSCI. The latter could provide coarse assessment of the perfusion borders [41,70], whereas the former could deliver assessable spatial resolution, imaging depth, and lateral blood vessels localization. Furthermore, OCT with angiography mode would allow visual assessment of both the structure and the microcirculation of the intestinal wall, with an option of visualizing vessels in the submucosal layer. Note that, such attribute has major significance in the planning of organ preservation surgery. The ability to obtain the combination of structural and angiographic data is the key characteristic distinguishing MM OCT from other similar diagnostic technologies.

Using MM OCT, we managed to achieve a high quality of visualization of the complex of serous and muscle layers and the vascular network localized within them. It should be emphasized, that useful and clinically significant results of the study of muscle fibers have previously been demonstrated by high-frequency ultrasound scanning of the rectal muscular layer [71]. However, CP OCT technology is an improvement as it offers imaging of individual muscle bundles and intermuscular spaces with higher resolution and contrast. The possibilities of their use for trans-serous intestinal studies have not yet been investigated. Other currently used optical methods for microcirculation imaging in the gastrointestinal tract (for example, SDF, LDF, NIR resonance spectrometry), as a rule, allow assessing changes in perfusion, but do not directly verify necrotic tissue [32]. Due to the irregular sensitivity of the layers of the intestinal wall to ischemia, blood vessels can remain in one layer when necrosis of another has already developed. Therefore, the ability of OCT to blood flow detection simultaneously with a tissue microstructure (determination of its viability or necrosis) is an important diagnostic advantage.

Along with the quality of the data obtained, the required duration of the investigative procedure is an important factor from a clinical point of view. In our pilot study [72], we have established that it takes only 8 to 12 min to obtain the 5–8 MM OCT images that are required to verify the depth of ischemic injury. In other words, the time required for MM OCT is only 6–10% of the total duration of the typical AMI operation, thereby significantly minimizing the period of anesthesia requirement and “open abdominal” state.

Our experience of experimental and clinical research has shown that the data obtained on the AMI model in laboratory animals could only partially be extrapolated to clinical diagnosis in patients. OCTA of the small intestine of patients has shown both similarities and differences from the data obtained in experiment on laboratory animals (rats, miniature pigs) [49,59]. Apparently, the differences in the OCTA picture of the small intestine (both in the norm and in ischemia) between laboratory animals and humans are due to the anatomical and physiological parameters of the vascular system of the small intestine, namely, the thickness and optical density of the serous membrane, the depth of the location of large vessels, their diameter and the blood flow rate. In an experiment on animals [49], we have previously identified diagnostically significant criteria for the preservation of intramural intestinal blood flow: 1—the presence of large “treelike”, often paired, vessels with a diameter of 50–200 μm; 2—a uniformly light background to the OCTA image, the presence of which depends on the functioning of the arterioles, venules, and capillaries of the submucosa and mucosa. In the OCTA images of an ischemic but viable human intestine, these criteria were met in no more than 70% of observations. In the present study, out of a total of 60 OCTA images obtained from 18 patients, 15 (25%) of them contained significant artefacts: Light stripes caused by intestinal peristaltic waves; the patient’s breathing movements; or pulse vibrations of the abdominal aorta. It is known that in other bioimaging methods used for angiography of the intramural bed of the intestine, peristaltic bowel movements are also the cause of rejection of 10–15% of images [21].

Group of Ton G. van Leeuwen (University of Amsterdam, The Netherlands) used a commercially available OCT system for detection of blood flow of gastric tissue after esophagectomy in order to reduce post-operative complications due to impaired perfusion [58]. The results showed the feasibility of intra-operative OCT-based imaging of gastric tissue and detection of flow in blood vessels in patients with esophageal cancer. 3D OCT data were obtained, but microcirculatory was demonstrated as blood vessel locations in structural B-scans. The idea was to distinguish areas with flow from static tissue, which became possible after speckle contrast (the ratio of speckle variance over the mean) calculation from OCT M-mode scans. The percentage of pixels distinguished as flow was quantified as an objective parameter that can potentially be used to differentiate normal from decreased perfusion areas. In this study, the focus was placed on speckle contrast to show blood flow, while the structural images themselves were not analyzed. The authors faced with the problem of motion artefacts induced by the surgeon’s hand as well as the patient’s heartbeat and breathing, therefore they failed to visualize blood vessel in the 3D OCT. It was also emphasized that quality of the images was suboptimal, as stabilization of the OCT probe was very difficult.

Building on the impressive result of [58], we were the first to demonstrate en-face 2D images of blood vessels network in normal and ischemic human intestine obtained in vivo during extempore surgery [72]. OCTA images were shown without any post-processing like those presented in the current work. This has been done in order to show the native images and artefact problems that are present when obtaining OCTA images in a real clinical setting. Of course, post-processing can significantly improve the OCTA image quality for the visualization of the microcirculatory network, but as practice shows, it can also result in loss of some important information about the vessels. Thus, together with the artefacts, vessels of a certain diameter may also be removed from the image, thereby impairing the reliability of the information. In this work, the aim was not to provide accurate quantification of the vascular networks. Therefore, presenting the material without post-processing is acceptable. In such cases, the vascular networks can be traced as brighter linear structures with clear outlines against a background of various kinds of noise.

Only one study was found, which presents the results of OCT imaging of the gastric wall structure from the side of the serous membrane [58]. The emphasis was placed on the blood vessels detection in OCT images, while OCT features in norm and ischemia were not described in detail. Prior to the current research, OCT visualization of the intestinal wall was not performed trans-serosally. We believe the reason is, primarily, insufficient scanning depth for observing the mucous membrane, including the villi, from where irreversible changes begin in ischemia. When studying ischemic injury, the attention of researchers is usually focused on the processes occurring in the mucous membrane. In our study, we showed that structural imaging of the outer layers up to the submucosa, combined with the microvascular network visualization, is sufficient to understand the depth of tissue damage.

The advantage of the used MM OCT setup is the presence of a cross-polarization channel. It provides a more contrasting visualization of the intestinal layers: The OCT signal is enhanced from polarization-sensitive structures (collagen and muscle fibers which is well organized in space) while it is weakened from structures that do not have such properties, only backward scattering. Thus, multimodality OCT imaging with polarization sensitivity is a promising instrument for intraoperative assessment of bowel viability.

There are some limitations of the study, which needs mentioning. Firstly, only a relatively small number of patients (18 persons) were enrolled; therefore, the result obtained at this stage is, essentially, a feasibility study to assess the information content and safety of MM OCT in the emergency diagnosis of human small intestine pathology. The final formulation of MM OCT diagnostic criteria for bowel pathology requires further accumulation of clinical data. Secondly, the MM OCT results in the control group cannot be fully accepted as a standard for intact human small intestine. Structural and functional changes in the “histologically intact” intestinal segments in patients with colorectal cancer are caused by epigenetic, microbiological, and biochemical factors; thus their long-term influence could have caused changes in the microstructure of the tissues and, accordingly, in the OCT picture of the ileal wall. However, based on the data presented, it can be argued that the presence of characteristic microstructural changes in the intestinal wall caused by acute ischemic injury (AMI group) is clearly contrasted with the intestine not damaged by AMI (control group). Therefore, at this stage of research it would be premature to recommend the routine use of MM OCT as a self-contained method for assessing the viability of sections of ischemic bowel. However, after elimination of limitations described above, the method could become one of the main techniques for the clinical diagnosis of the extent of intestinal tissue damage in AMI.

Additional limitations of the study are as follows. The MM OCT is the most effective in solving specific problems in a certain group of patients with AMI, but not in all patients with impaired blood flow of intestine. In situations when the entire damaged area with approximate boundaries should be identified in the shortest period of time, LSCI or FI are more optimal methods. Our assessment suggests that MM OCT data played the most significant role in specific clinical situations. Several clinical examples are demonstrated below. In case of polysegmental focal bowel infarction, spread over 320 cm of its length in the basin of the second and third segments of the superior mesenteric artery, MM OCT helped to avoid unnecessary expansion of the resection boundaries and to remove only confluent lesions. In another patient with a history of gastric resection and widespread adhesions in the upper abdominal cavity, the presence of perfused vessels and normal microstructure found by MM OCT in the visually altered intestine helped to preserve a proximal segment of intestine sufficient to form an anastomosis. In routine clinical cases in patients with severe abdominal obesity, MM OCT data allows quick and more accurate determination of tissues with preserved perfusion due to the perfused intramural vessels found by OCTA. The use of MM OCT appears to be promising for the diagnosis of intestinal damage against the background of microthrombosis of intramural and terminal mesenteric vessels. This feature of ischemic damage is one of the common COVID-19 infection complications [73]. However, we do not yet have data on the diagnosis of mesenteric ischemia in COVID-19 patients using MM OCT.

It should be noted that at the present stage we do not consider MM OCT as an alternative to the methods of screening assessment of the presence of blood circulation over tens or hundreds of centimeters of ischemic bowel. To resolve routine diagnostic tasks, it is advisable to use techniques that have proven efficacy: LSCI [41,70] or fluorescence angiography [19,20]. However, in some clinical situations, accurate topical diagnosis of perfused vessels is required, which can only be performed using MM OCT. In particular, MM OCT could be crucial in areas bordering the damaged intestine, in situations when it is necessary to resect as economically as possible particularly in patients with subtotal intestinal injury, or when it is necessary to preserve small, but physiologically important, areas of the intestine such as the terminal ileum or the initial parts of the jejunum. The unique possibility of simultaneous assessment of microcirculation and microstructure using MM OCT increases the information capacity of the study in a group of patients with intestinal hypoperfusion caused by general hypotension or a side effect of inotropic drugs. In these cases, the microstructure of the layers of the intestinal wall could become an important criterion for choosing the boundaries of bowel resection.

## 5. Conclusions

MM OCT allows simultaneous assessment of structure and microcirculation with high resolution, which is crucial for clinicians in defining the boundaries of bowel resection in AMI. Intraoperative MM OCT performed by trans-serous access is a potential diagnostic tool for objective assessment of the condition of intestinal wall layers along with the vessels localized within them. The MM OCT images of non-ischemic, ischemic, viable, and necrotic small intestine differ significantly in the volume of extravasal fluid accumulation, the structure of muscle bundles in the longitudinal muscle layer, and the type and density of the vasculature.

The MM OCT diagnostic procedure optimally meets the requirements of emergency surgery as it requires relatively less time to examine the microstructure and microcirculation of the intestine along its entire length from the direction of the serous membrane without resorting to transluminal interventions and contrasts. Optionally, the MM OCT data can include objective quantitative parameters. Despite the advantages, MM OCT method of data acquisition requires significant improvement in order to reduce the number of motion artefacts. In actual clinical conditions it may not be as effective as in a laboratory condition. Application on animals in a controlled laboratory environment allows for maximal stabilization of the moving system parts and the studied organ which is near impossible to replicate under actual clinical condition.

## Figures and Tables

**Table 1:** Characteristics of the patient groups.

Characteristic	AMI Group	Control Group	*p* Value
Sex (male/female), *n*	6/3	4/5	0.095 *
Median age, years, Me [Q1;Q3]	67 [62; 73]	62 [57; 69]	0.190 **
Median body mass index, Me [Q1;Q3]	24.8 [23.4; 26.8]	27.3 [26.2; 27.7]	0.113 **
Smoker, *n*	3	2	0.647 *
Diabetes, *n*	1	2	0.602 *
Hypertension, *n*	6	5	0.667 *
Chronic obstructive pulmonary disease, *n*	1	0	-
Abnormal heart rhythm, *n*	4	2	0.376 *

* Fisher’s exact test; ** the Mann–Whitney test.

**Table 2:** Hemodynamic and blood parameters during MM OCT examination, Me [Q1; Q3].

Parameter	AMI Group	Control Group	*p* Value (the Mann-Whitney Test)
Hemodynamics			
Heart rate, beats/min	78 [72; 87]	82 [72; 91]	0.489
Systolic arterial pressure, mm Hg	118 [110; 127]	128 [118; 135]	0.605
Diastolic arterial pressure, mm Hg	62 [55; 77]	67 [60; 72]	0.546
Mean arterial pressure, mm Hg	80 [75; 93]	84 [80; 90]	0.666
Abdominal perfusion pressure	72 [65; 87]	79 [73; 84]	0.678
Central venous pressure, mm Hg	6 [3; 10]	8 [4; 10]	0.546
Peripheral oxygen saturation, %	98 [97; 99]	98 [96; 99]	0.730
Body temperature, °C	36.5 [36.4; 36.9]	36.5 [36.2; 36.8]	0.796
Blood			
Le, ×10^9^/L	12.0 [10; 16.5]	9.2 [8.4; 11.1]	0.063
Er, ×10^12^/L	4.1 [3.5; 5.2]	4.1 [3.8; 4.7]	0.863
Hb, g/L	131 [124; 141]	123 [111; 137]	0.258
Protein, g/L	65 [63.3; 73.3]	69.4 [62; 77.5]	0.796

**Table 3:** Characteristics of MM OCT features of the small intestine in studied groups of patients. *n*-number of regions of interest (ROI).

Group of ROI		Structural OCT Features						Angiographic OCT Features		
		Detectable Layers of Small Intestine, *n*-Abs. (%)					Inter-Muscular Spaces	Subserosal Blood Vessels	Micro- Vascular Network	OCTA Description
		Serosa	Outer Mus- cular Layer	Inner Mus- cular Layer	Sub Mucosa	Mucosa				
Control Group (*n* = 18)		18 (100)	18 (100)	18 (100)	12 (66.7)	2 (11.1)	Narrow slit-like spaces between the muscle bundles localized of only within the outer muscular layer	yes	yes	Multiple branched blood vessels anastomosed with each other, the background is light
AMI Group	Ischemia(*n* = 18)	18 (100)	18 (100)	18 (100)	6 * (33.3)	-	Wide numerous spaces localized between the muscle bundles of the both muscular layers, as well as at the borders of the serous and outer muscular layers and of the outer and inner muscular layers	yes	partly	Sparse vasculature or single, predominantly non-anastomosed blood vessels with each other, the background is mostly dark
	Necrosis (*n* = 9)	4 (44.4)	-		2 * (22.2)	-	no	no	no	Scraps of single blood vessels or their complete absence, the background is dark

* Only the boundary between the inner muscular layer and submucosa was distinguished.

## Data Availability

The data presented in this study are available on request from the corresponding author.
